# Inc/GFP chimera protein-based interactomics reveals host cellular interactions of Cps0558, a novel *Chlamydia psittaci* inclusion protein

**DOI:** 10.1093/femspd/ftaf012

**Published:** 2025-10-22

**Authors:** Jean-Marc Gensch, Jana Scholz, Alyssa Ingmundson, Laura Rose, Joerg Doellinger, Sebastian Banhart, Dagmar Heuer

**Affiliations:** Unit of Sexually Transmitted Bacterial Pathogens and HIV, Robert Koch Institute, 13353 Berlin, Germany; Unit of Sexually Transmitted Bacterial Pathogens and HIV, Robert Koch Institute, 13353 Berlin, Germany; Unit of Sexually Transmitted Bacterial Pathogens and HIV, Robert Koch Institute, 13353 Berlin, Germany; Unit of Sexually Transmitted Bacterial Pathogens and HIV, Robert Koch Institute, 13353 Berlin, Germany; Unit of Proteomics and Spectroscopy (ZBS6), Robert Koch Institute, 13353 Berlin, Germany; Unit of Sexually Transmitted Bacterial Pathogens and HIV, Robert Koch Institute, 13353 Berlin, Germany; Unit of Sexually Transmitted Bacterial Pathogens and HIV, Robert Koch Institute, 13353 Berlin, Germany

**Keywords:** *Chlamydia psittaci*, *Chlamydia*, inclusion membrane proteins, chimera, host-pathogen interaction, interactomics, proteomics, intracellular bacteria

## Abstract

The obligate intracellular Gram-negative bacterium *Chlamydia psittaci*, a zoonotic pathogen transmissible between birds and humans, has played a pioneering role in research on its membrane-bound replicative niche termed the inclusion. Inclusion membrane proteins (Inc proteins) are crucial for *Chlamydia*–host interactions and were first identified in *C. psittaci*. This study investigates putative *C. psittaci* Inc proteins by a combination of *in silico* analyses, immunofluorescence and, strikingly, a new Inc/GFP chimera protein-based interactomics approach to identify host cellular interaction partners. Here, we report a novel *C. psittaci* Inc protein, Cps0558, along with respective host cellular interaction partners, in particular ACAD11, which is involved in lipid metabolism. We confirm their physical interaction in the native infection context, supporting the physiological relevance of our chimera-based screen. Furthermore, new interaction partners for the known Inc protein IncA are identified, revealing a potential role of IncA as modulator of the host ubiquitylation system. These results provide further insights into the biology of *C. psittaci* and present a novel tool for studying Inc proteins under conditions closely resembling their natural tertiary structure.

## Teaser

This study investigates putative *Chlamydia psittaci* Inc proteins by a combination of *in silico* analyses, immunofluorescence, and a new Inc/GFP chimera protein-based interactomics approach to identify host cellular interaction partners.

## Introduction

The Gram-negative bacterium and obligate intracellular pathogen *Chlamydia psittaci* (Moulder 1985), a member of the *Chlamydiaceae* family, has a significant impact on both avian and human health (Bayramova et al. 2018). *Chlamydia psittaci* can infect birds as its natural host and has the potential for zoonotic transmission to humans by inhalation of infectious aerosols, resulting in psittacosis (Vanrompay et al. 1995). In humans, *C. psittaci* infection can cause typical pneumonia and—if left untreated—can lead to acute sepsis (Knittler et al. 2014, Knittler and Sachse 2015, Radomski et al. 2016). Notably, *C. psittaci* is adapted to a diverse range of hosts including parrots, pigeons, swine (Vanrompay et al. 2004), and cattle (Kemmerling et al. 2009), and shares a genomic lineage with other rather host-specific *Chlamydia* species (Hölzer et al. 2020), such as the relevant sexually transmitted human pathogen *Chlamydia trachomatis* (O’Connell and Ferone 2016). *Chlamydia psittaci* lacks orthologous genes that were found in *C. trachomatis* such as IncE, which recruits the retromer complex by binding to SNX5/6 (Mirrashidi et al. 2015, Paul et al. 2017) and IncG, which interacts with 14–3-3β to modulate host apoptotic signaling (Scidmore and Hackstadt 2001, Verbeke et al. 2006); nevertheless, it has successfully adapted to infect humans, making it a zoonotic pathogen. This ability to infect both birds and humans highlights the fundamental survival mechanisms that enable *Chlamydia* species to adapt to different hosts. Studying *C. psittaci* as a zoonotic pathogen can therefore provide critical insights into the core mechanisms required for chlamydial survival and adaptation, offering valuable information for the development of prevention and treatment strategies targeting *Chlamydia* infections.

The biphasic developmental cycle of *Chlamydia* spp. involves alteration between two distinct developmental forms: infectious elementary bodies (EBs) and noninfectious reticulate bodies (RBs) (Tamura and Manire 1967, Lawn et al. 1973, Moulder et al. 1980). Initially, EBs attach to the host cell (Su et al. 1996, Su and Caldwell 1998, Stephens et al. 2001, Taraktchoglou et al. 2001, Fadel and Eley 2008), preferentially epithelial cells (Maslow et al. 1988, Johnson and Kerr 2015), where they inject effector proteins through the type 3 secretion system (T3SS), ultimately leading to endocytosis (Clifton et al. 2004, Saka et al. 2011). Subsequently, bacteria develop within a membrane-bound compartment, called inclusion (Belland et al. 2003). In the early developmental phase between 2 and 8 h postinfection (p.i.), EBs differentiate into the replicative RB form (Christensen et al. 2019) and *de novo* protein synthesis of bacterial proteins is initiated (Shaw et al. 2000). Characteristic for members of the *Chlamydiaceae* is their development in the inclusion, which is build up by integral Inclusion membrane proteins (Inc proteins). Inc proteins are translocated into the inclusion membrane via T3SS (Rockey et al. 1995), where they can interact with host cellular proteins (Scidmore and Hackstadt 2001, Rzomp et al. 2006, Delevoye et al. 2008). During the mid-phase between 8 and 24 h p.i. (Scholz et al. 2024), Inc proteins recruit specific cellular proteins and organelles to the growing inclusion, thereby altering host cell processes to create an environment beneficial for chlamydial replication (Bugalhão and Mota 2019). Subsequently, in the late phase of the developmental cycle, RBs redifferentiate into EBs, which ultimately egress to initiate another round of infection (Belland et al. 2003, AbdelRahman and Belland 2005).

Inc proteins play a central role in mediating interactions between *Chlamydia* spp. and the host cell. One characteristic feature of these proteins is their bi-lobed structure including two transmembrane (TM) domains (Rockey et al. 1995). However, exceptions like the *C. trachomatis* Inc protein IncV with four TM α-helices exist (Stanhope et al. 2017). Inc proteins expose their terminal domains to the host cytosol, where they can interact with the host and show diverse functions. The first identified Inc protein, IncA, was discovered in *C. psittaci* (Rockey et al. 1995), followed by IncB and IncC (Bannantine et al. 1998). While orthologs are present across various species, their sequence similarity remains relatively poor (Dehoux et al. 2011, Lutter et al. 2012). Such orthologues are called core Inc proteins like IncB and IncC, which have conserved characteristics but differ regarding their interaction partners. For example, IncB from *C. psittaci* interacts with human Snapin, a crucial contributor to intracellular trafficking and the microtubule network (Böcker et al. 2014), while the interactions of IncB from *C. trachomatis* remain still unknown (Mirrashidi et al. 2015). Notably, *C. trachomatis* IncA participates in homotypic fusion of chlamydial inclusions (Ronzone and Paumet 2013) and interacts with VAMP3/7/8 (Delevoye et al. 2008), whereas IncA of *C. psittaci* interacts with Ras-GTPase activating protein SH3 domain-binding protein 1 (G3BP1) (Borth et al. 2011). Although the first description of an Inc protein was published for *C. psittaci*, the majority of literature focusses on Inc proteins from *C. trachomatis*. To date, *C. trachomatis* is known to encode at least 36 bona fide Inc proteins, which have provided significant insights into the pathogen’s complex interactions with the host cell (Bugalhão and Mota 2019). In contrast, research on *C. psittaci* Inc proteins has progressed more modestly, with only seven bona fide Inc proteins experimentally confirmed so far (Rockey et al. 1995, Bannantine et al. 1998, Wu et al. 2016, He et al. 2021, Tang et al. 2021, Huang et al. 2023, Xiao et al. 2023).

Investigation of protein function and localization via genetic manipulation of *Chlamydia* has long been technically challenging due to its obligate intracellular lifestyle and unique developmental cycle (Tam et al. 1994, Heuer et al. 2007). Nevertheless, substantial progress has been made for *C. trachomatis*, with the establishment of plasmid-based transformation systems, targeted mutagenesis, allelic exchange mutagenesis, and CRISPR interference (Wang et al. 2011, Johnson and Fisher 2013, Mueller et al. 2016, Weber and Faris 2019, Ouellette et al. 2021). These tools have enabled functional studies of chlamydial genes and host–pathogen interactions. Notably, the use of APEX2-tagged Inc proteins for proximity labeling has yielded detailed interactome maps of the inclusion membrane (Rucks et al. 2017, Dickinson et al. 2019). Although stable expression of fluorescent proteins has recently been achieved in *C. psittaci* (Shima et al. 2020), broader application of genetic manipulation strategies to this species is still missing. This gap may reflect practical and regulatory barriers, including biosafety restrictions for the genetic engineering of zoonotic human pathogens. To overcome these limitations and enable the study of *C. psittaci* inclusion membrane proteins, we developed an alternative strategy based on Inc/GFP chimera protein-based interactomics. This method offers a rapid and genetically independent approach for investigating Inc protein localization and interactions.

In this study, we aim to identify and characterize novel *C. psittaci* Inc proteins. We employed *in silico* analysis and immunofluorescence staining to identify *C. psittaci* Inc proteins. Using a novel method, which relies on Inc/GFP chimera protein-based interactomics, we were able to describe host cellular interactions of Cps0558, a novel *C. psittaci* inclusion protein, and IncA. In addition, using this systematic and comprehensive approach we lay the groundwork for similar investigations in other intracellular pathogens.

## Materials and methods

### Reagents and antibodies

Unless otherwise stated, all reagents were obtained from Sigma-Aldrich. Cell culture media and supplements (RPMI 1640, DMEM, phosphate-buffered saline (PBS), sodium pyruvate, and l-glutamine) were obtained from GIBCO; fetal calf serum (FCS) was purchased from Sigma-Aldrich. Polymerase chain reactions (PCRs) were performed with Phusion High-Fidelity PCR Master Mix with HF Buffer (Thermo Scientific) and oligonucleotides obtained from BioTeZ. Enzymes for cloning were derived from NEB (AscI, NotI-HF, Antarctic phosphatase) and Thermo Scientific (T4 DNA Ligase). The following antibodies were used for immunofluorescence staining and/or western blotting: mouse antichlamydial HSP60 (1:500; catalog number MA2-023; Thermo Fisher Scientific, Bremen, Germany); rabbit anti-*C. trachomatis* IncE (1:100; WB 1:500; Rose 2021); rabbit anti-*C. psittaci* IncA (Scholz et al. 2024); mouse anti-c-myc (9E10) (1:100; catalog number sc-40; Santa Cruz Biotechnology); mouse anti-ACAD11 (1:50; catalog number H00084129-A01; Abnova); mouse anti-ACAD11 (WB 1:200; catalog number sc-514027; Santa Cruz Biotechnology); Alexa Fluor 488-coupled goat antirabbit IgG (1:100; catalog number 111–545-144; Dianova); Cy3-coupled goat antimouse IgG (1:200; catalog number 115–165-146; Dianova); rabbit anti-SNX5 (WB 1:500; catalog number ab180520; abcam); rabbit-anti-GFP (WB 1:5000; catalog number A-6455; Invitrogen); HRP-conjugated goat antimouse IgG (WB 1:5000; catalog number 926–80 010; Li-Cor Bioscience), and HRP-conjugated goat antirabbit IgG (WB 1:5000; catalog number 926–80 011; Li-Cor Bioscience).

### Cloning of Inc/GFP chimera proteins and GST–Inc expression plasmids

We used a combination of extension-PCR, overlap-PCR (Higuchi et al. 1988, Hilgarth and Lanigan 2020), and classic restriction site digestion and ligation to generate the plasmids for Inc/GFP chimera proteins expression and expression of GST–Inc fusion proteins for antibody production. The Inc/GFP chimera protein construct was generated from three fragments: the N-terminal cytosolic domain of an Inc protein, eGFP, and the C-terminal cytosolic domain of the same Inc protein, flanked by AscI and NotI restriction sites. We extended these fragments with overlapping sequences by extension PCR. Two fragments with a matching overlap were used in one overlap-PCR. Subsequently, we combined the product with the third fragment in another overlap-PCR. This final insert was cloned into pEGFP-C1 (Clontech) using AscI and NotI restriction sites. To produce the GST–Inc fusion protein expression vector for antibody generation, we flanked the C-terminal domain of an Inc protein with AscI and NotI, and cloned this insert into pGEX-3X N-terminal *Schistosoma japonicum* glutathione *S*-transferase (GST) expression vector (GE Healthcare).

### Antibody production

The polyclonal rabbit antibody against Cps0558 was produced by immunization of rabbits with the C-terminal cytosolic domain of Cps0558 fused to GST. All animal handling and antibody purification was performed by Biogenes, Berlin. The antigen was produced in *Escherichia coli* Rosetta 2 (Merck) transformed with the GST–Inc fusion protein expression vectors described. Expression of GST–Inc fusion proteins was induced by addition 1 mM Isopropyl β-d-1-thiogalactopyranoside (IPTG, Roth) to LB medium with 50 µg ml^−1^ ampicillin. Protein expression was performed at 37°C for 4 h. Subsequently, pelleted bacteria were incubated with lysis buffer [50 mM Tris, 0.1% Triton X100, 100 µg ml^−1^ lysozyme, 500 U µl^−1^ nuclease, pH 8.0, with Complete Protease Inhibitor Cocktail (Sigma Aldrich)] on ice, followed by sonification and centrifugation. Soluble GST–Inc fusion protein was bound to Glutathion-Sepharose 4B (Sigma-Aldrich) in a 5 ml column (Thermo Scientific) and washed with PBS. The GST–Inc fusion protein was eluted using elution buffer (50 mM Tris–HCl, 10 mM reduced glutathione, pH 8.0) at room temperature (RT) for 20 min, repeated three times.

### Cell culture, infection, and transfection

HeLa cells (ATCC-number CCL-2) were cultivated in RPMI 1640 medium [10% (v/v) heat-inactivated FCS, 1 mM sodium pyruvate, 2 mM l-glutamine] at 37°C and 5% (v/v) CO_2_ in a humidified incubator and passaged every 2–3 days. *Chlamydia psittaci* strain 02DC15 genotype A-VS1 (cattle isolate, 2002, kindly provided by Konrad Sachse, Institute of Molecular Pathogenesis, Friedrich-Loeffler-Institut, Jena, Germany) was propagated in and purified from HeLa cell monolayers. Infections were performed in Dulbecco’s modified Eagle’s medium (DMEM) [5% (v/v) FCS, 4.5 g l^−1^ glucose, 1 mM sodium pyruvate, 2 mM l-glutamine] at 35°C and 5% (v/v) CO_2_ in a humidified incubator. Cells were infected with *C. psittaci* as follows: After washing with infection medium, cells were incubated with bacteria using the indicated multiplicity of infection (MOI) for initial 30 min. Subsequently, infections were centrifuged at 600 × *g* for 30 min, followed by additional incubation for 60 min until the inoculum was replaced with fresh DMEM. For plasmid transfection of HeLa cells, cells were grown to a confluency of 80%. The transfection was performed with Lipofectamine 2000 reagents (Invitrogen) according to manufacturer’s protocol.

### Generation of SNX5/6-KO cells using CRISPR/Cas9

We generated stable knockout cell lines using the CRISPR/Cas9 system. Herein, we designed the single guide RNA (sgRNA) sequences targeting the Cas9 to the specific genomic locus according to Ran et al. (2013). The sgRNA sequence (SNX5 KO: CTGCAGCAACTCGGGAACCG) was inserted into the expression vector pSpCas9n(BB)-2A-Puro (PX462) (Addgene #48141) using the restriction enzyme BsbI. Subsequently, we transfected the final sequence-verified plasmid into HeLa cells, selected them with 1.5 µg ml^−1^ puromycin (Carl Roth), and clonally cultivated isogenic cell lines from isolated cells. SNX5/SNX6 double KO was established using the SNX5 single KO cell line as background (SNX6 KO sgRNA sequence: AGAATTCACAAAGATGAAAC). Positive cell lines were validated by immunofluorescence, western blot analysis, quantitative polymerase chain reaction (qPCR), and mass spectrometry (MS) analysis (parallel reaction monitoring) as well as genomic sequencing of the target region.

### Immunofluorescence staining

Immunofluorescent staining was performed as previously published (Banhart et al. 2014). In brief, cells grown on glass coverslips were washed with PBS and fixed with 2% formaldehyde for 30 min. Fixation, blocking, permeabilization, and antibody incubation was performed at RT. The samples were blocked and permeabilized using 0.2% (v/v) Triton-X and 0.2% (w/v) bovine serum albumin (BSA) in PBS for 20 min. The antibodies were diluted in 0.2% (w/v) BSA in PBS. The cells were incubated with primary antibodies for 1 h, followed by PBS washing, and subsequent incubation with secondary antibody along with DAPI (1:5000) for 1 h. Finally, washed cells were mounted onto microscopy slides using Mowiol (Sigma Aldrich). Images were captured using a laser scanning confocal microscope (Carl Zeiss LSM 780; Leica Microsystems Stellaris 8). For three-dimensional analysis, z-stacks were acquired with step sizes optimized for inclusion volume coverage.

### Image analysis and colocalization quantification

Confocal microscopy images were analysed using Fiji (ImageJ 1.53f; Schindelin et al. 2012). For three-dimensional analysis of fluorescence localization, z-stacks were acquired using confocal microscopy and orthogonal projections (XY, XZ, and YZ) were generated in Fiji using the Orthogonal Views tool. For quantification of colocalization, the Coloc2 plugin was used to calculate Pearson’s correlation coefficient between the fluorescence signals of the two proteins of interest within manually selected regions of interest around inclusion membrane sections. Pseudo-coloring in green and magenta was applied for visualization, independent of actual laser excitation wavelengths. Statistical analysis and plots were done with GraphPad Prism 8.4.0 (GraphPad Software).

### Coimmunoprecipitation from infected cells

HeLa cells were infected with *C. psittaci* MOI 2 and harvested at 46 h p.i. Cells were washed with PBS and lysed in IP lysis buffer (10 mM Tris/Cl pH 7.5, 150 mM NaCl, 0.5 mM EDTA, 0.5% NP-40) supplemented with protease inhibitors (Roche cOmplete, EDTA-free). After incubation on ice for 25 min, lysates were clarified by centrifugation at 16 000 × *g* for 10 min at 4°C. The supernatants were incubated overnight at 4°C with the rabbit polyclonal antibody generated against Cps0558. After Pierce protein A/G magnetic beads (Thermo Fisher) were added, samples were incubated 40 min at 4°C then 40 min at room temperature. Beads were subsequently washed five times with lysis buffer and processed for SDS-PAGE and western blotting as described below.

### SDS-PAGE

Proteins were separated by SDS-polyacrylamide gel electrophoresis (SDS-PAGE) using hand-cast 10% polyacrylamide gels. Samples were prepared in 2x Laemmli buffer (250 mM Tris/HCl, 32% glycerol, 6% 2-mercaptoethanol, 4% SDS, 0.02% bromphenol blue, pH 6.8), denatured at 95°C for 10 min, and immediately loaded onto the gel alongside a prestained protein ladder (PageRuler™ Prestained Protein Ladder, Thermo Scientific). Electrophoresis was performed using a Mini-PROTEAN system (Bio-Rad), first at 70 V to allow stacking, followed by separation at 140 V until the dye front reached the bottom of the gel.

### Western blotting

Proteins were transferred onto a methanol-activated polyvinylidene fluoride membrane (Immobilon®-P, Millipore) using wet transfer at 200 mA for 2 h at 4°C in a Mini-PROTEAN Tetra system (Bio-Rad). Membranes were blocked for 1 h at 4°C in 3% milk in TBS-T (100 mM Tris/HCl, 1.5 mM NaCl, 0.05% Tween-20, pH 8.0), and incubated overnight at 4°C with the appropriate primary antibody diluted in blocking buffer. After three washes in TBS-T (10 min each), membranes were incubated for 1 h at room temperature with horseradish peroxidase (HRP)-conjugated secondary antibody. Signal detection was performed by enhanced chemiluminescence (ECL; Amersham™ ECL Western Blotting Detection Reagents, GE Healthcare), followed by exposure to X-ray films (Amersham Hyperfilm™ ECL). Films were developed using a CP1000 automatic film processor (Agfa-Gevaert), labeled, and scanned for documentation.

### Sample preparation for proteomics

For sample preparation, cells were transfected with respective Inc/GFP chimera protein plasmids, whole cell lysates were collected at 24 h posttransfection and lysates were subjected to GFP-Trap (Chromotek) using magnetic agarose beads for separation following the manufacturer’s instructions for the iST GFP-Trap Kit (Chromotek). After purification, the beads were incubated with urea solution (8 M urea, 2 M thiourea, 5 mM DTT, 50 mM Tris/HCl, pH 8.5) at 37°C for 1 h. Denatured and reduced proteins were then alkylated in final 15 mM 2-iodoacetamide at RT for 30 min. Samples were diluted 1:8.5 with 50 mM Tris/HCl (pH 8) and digested with 200 ng trypsin overnight at 37°C. Digestion was stopped by acidification with a final concentration of 2% (v/v) trifluoroacetic acid (pH 2). Precipitates were centrifuged and peptides desalted according to Rappsilber et al. (2007) via StageTips with 3x C18 discs (Empore) and concentrated by vacuum. Peptides were resuspended in 0.1% (v/v) formic acid, and concentration measured with NanoDrop 2000 (Thermo Scientific) at 280 nm (AU 1.1 µg^−1^).

### Liquid chromatography and mass spectrometry

Peptides were analysed on an EASY-nanoLC 1200 (Thermo Fisher Scientific) coupled online to a Q Exactive™ HF mass spectrometer (Thermo Fisher Scientific). Peptides were loaded on a Acclaim™ PepMap™ trap column (20 mm × 75 µm i.d., 100 Å, C18, 3 µm; Thermo Fisher Scientific) at a flow rate of 2 µl min^−1^ for 6 min and were subsequently separated on 200 cm µPAC™ column (PharmaFluidics, Ghent, Belgium) using a 100 min segmented gradient of 2%–33% acetonitrile in 0.1% formic acid at 300 nl min^−1^ flow rate. Column temperature was kept at 50°C using a butterfly heater (Phoenix S&T, Chester, PA, USA). The Q Exactive™ HF was operated in a data-dependent manner in the m/z range of 300–1650 with a resolution of 60 000 using an automatic gain control (AGC) target value of 3 × 10^5^ with a maximum injection time of 50 ms. Up to the 20 most intense 2+–5+ charged ions were selected for higher-energy c-trap dissociation with a normalized collision energy of 25%. Fragment spectra were recorded at an isolation width of 2 Th and a resolution of 15 000 at 200 m/z using an AGC target value of 1 × 10^5^ with a maximum injection time of 25 ms. The minimum MS^2^ target value was set to 1 × 10^4^. Once fragmented, peaks were dynamically excluded from precursor selection for 30 s within a 10 ppm window. Peptides were ionized using electrospray with a stainless-steel emitter, I.D. 30 µm, (Proxeon, Odense, Denmark) at a spray voltage of 2.1 kV and a heated capillary temperature of 275°C.

### Data analysis

The mass spectra were analysed using MaxQuant (version 1.6.5). At first, parent ion masses were recalibrated using the “software lock mass” option before the MS^2^ spectra were searched using the Andromeda algorithm against the human proteome obtained from UniProt (UP000005640, downloaded 23 May 2019) and either the *C. psittaci* 02DC15 proteome obtained from NCBI or the *C. trachomatis* (strain D-UW-3-Cx) proteome obtained from UniProt (UP000000431, downloaded 31 August 2016). Spectra were searched with a tolerance of 4.5 ppm in MS^1^ and 20 ppm in MS^2^ mode, strict trypsin specificity (KR not P) and allowing up to two missed cleavage sites. Cysteine carbamidomethylation was set as a fixed modification and methionine oxidation as well as N-terminal acetylation of proteins as variable modifications. The false discovery rate was set to 1% for peptide and 1% protein identifications. Identifications were transferred between samples using the “match between run” option within a match window of 0.7 min and an alignment window of 20 min. For investigation of protein interaction partners after GFP-Trap and LC–MS, the identified proteins underwent Significance Analysis of INTeractome (SAINT) enrichment analysis for significance based on spectral count comparison among all samples (Choi et al. 2011, Teo et al. 2014, Olson et al. 2020). We declared proteins with a SAINT score ≥0.9 and a Bayesian false discovery rate (BFDR) <0.01 to be significantly enriched. Graphs for visual data presentation were generated by GraphPad Prism 8.4.0 (GraphPad Software).

### Bioinformatics

For the initial *in silico* analysis, we analysed the preannotated genomes of *C. psittaci* 02DC15 (accession number CP002806.1), *C. psittaci* VS225 (accession number CP03793.1), and *C. psittaci* 6BC (accession number CP002549.1). Term screening, identification of orthologous sequences among genomes, and sequence alignments were performed with the software Geneious 11.1.5 (Biomatters Development Team) and the web tool Clustal Omega (Madeira et al. 2019). Based on amino acid (aa) sequences, we predicted TM domains using Protter (Omasits et al. 2014) and analysed the hydrophobicity using the Kyte & Doolittle score with ProtScale Hydrophobicity Tool from Expasy (Gasteiger et al. 2005). For comparison of protein structures, we used PyMOL (The PyMOL Molecular Graphics System, Version 2.0 Schrödinger, LLC). Herein, we compared the structure of eGFP (PDB: 2Y0G) with the structure of IncE from *C. trachomatis* serovar D, which was predicted by AlphaFold (Jumper et al. 2021, Varadi et al. 2021) based on the UniProt ID P0DJI4.

## Results

### *In silico* analyses identify seven uncharacterized putative *C. psittaci* Inc proteins

**Figure 1.**
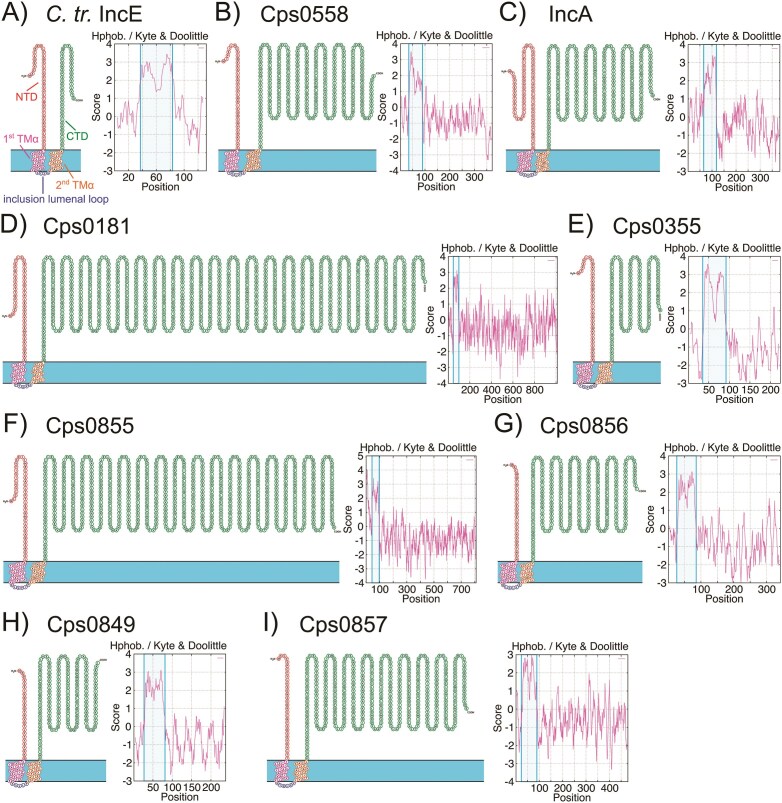
*In silico* prediction of protein topology and TM domains for novel putative *C. psittaci* Inc proteins. The aa sequences of the positive control *C. trachomatis* (*C. tr*.) IncE (A) and the identified *C. psittaci* Inc proteins Cps0558 (B), IncA (C), Cps0181 (D), Cps0355 (E), Cps0855 (F), Cps056 (G), Cps0849 (H), and Cps0857 (I) were used to predict protein topology and TM domains using both Protter (left) and Kyte & Doolittle scale calculation (right). Different Inc protein domains are shown in the Protter view: the cytosolic N-terminal domain (NTD), first TM α-helix (1st TMα), inclusion luminal loop, second TM α-helix (2nd TMα), and cytosolic C-terminal domain (CTD). The inclusion membrane separates the upper compartment (host cytosol) from the lower compartment (inclusion lumen). The predicted TM domains, including the luminal loop, are highlighted by frames in the hydrophobicity plots.

The current lack of experimentally validated *C. psittaci* Inc proteins is impeding investigation and understanding of the biology of this intracellular pathogen. We therefore aimed to identify novel, uncharacterized *C. psittaci* Inc proteins by *in silico* analyses. First, we selected all open reading frames from the genome of *C. psittaci* 02DC15 (accession number CP002806.1), which is widely used in experimental studies (Koch-Edelmann et al. 2017, Scholz et al. 2024) and aligned them with the well-annotated genome of *C. psittaci* VS225 (accession number CP037931.1), focusing on genes annotated with terms indicative of Inc proteins or poorly characterized genes (e.g. “putative,” “hypothetical,” “conserved membrane protein,” “inclusion membrane protein,” “IncA family protein,” “IncB,” or “IncC”). For broader context, we also compared these genes with the genome of *C. psittaci* 6BC (accession number CP002549.1), a commonly used reference strain in experimental research (He et al. 2021, Tang et al. 2021). All identified coding sequences were subsequently screened for TM domains using Protter (Omasits et al. 2014). Based on the typical bi-lobed membrane topology of Inc proteins, we selected candidates with two predicted TM domains (Table S1). Proteins from *C. psittaci* 02DC15 were abbreviated as CpsXXXX, based on their locus tags CPS0B_XXXX. In total, we identified 60 putative *C. psittaci* Inc proteins, of which 47 had predicted orthologs in *C. caviae* (Dehoux et al. 2011), and 6 in *C. trachomatis* (Pereira et al. 2024). To refine our dataset, we prioritized proteins that were computationally preannotated as Inc proteins (highlighted in yellow). Our subsequent analysis focused on these 11 putative Inc proteins (Table 1), including the four already described Inc proteins IncA, IncB, IncC, and Cps0850 (Huang et al. 2023, Xiao et al. 2023). Notably, Cps0850 is unrelated to *C. trachomatis* CT850 despite the similar nomenclature. The remaining seven identified putative Inc proteins were unknown and uncharacterized: Cps0558, Cps0857, Cps0855, Cps0856, Cps0181, Cps0355, and Cps0849 (Table 1). These proteins varied in length between 184 and 1012 aa with a median length of 352 aa. Notably, there were two large proteins with 1012 aa (Cps0181) and 810 aa (Cps0855) (Table 1). To test whether the identified proteins shared structural characteristics of already described Inc proteins, we visualized the predicted potential TM domains using Protter (Omasits et al. 2014). Results were further validated by determining the score-based hydrophobicity of the aa sequences according to Kyte & Doolittle (Gasteiger et al. 2005). During the whole study, we used the well characterized Inc protein IncE of *C. trachomatis* as positive control. IncE consists of an N-terminal cytosolic domain (red), followed by two TM α-helices (pink and orange) separated by an inclusion luminal loop region (blue) and a C-terminal cytosolic domain (green) (Fig. 1A). Comparable to IncE (Fig. 1A), all putative Inc proteins shared the same domain organization (Fig. 1B–H). The loop regions were all similar in length, ranging from five to six aa (Fig. 1, Table 1). Interestingly, C- and N-terminal regions differed in length among the seven putative Inc proteins (N-terminus: 26–48 aa, C-terminus: 134–918 aa) (Fig. 1, Table 1), suggesting a varying potential to interact with host cellular proteins. In addition, all hydrophobicity plots identified highly hydrophobic regions consistent with the predicted TM domains (Fig. 1, Fig. S1). Taken together, these findings support that the seven *in silico* predicted proteins represent novel *C. psittaci* Inc proteins.

**Table 1.** Overview of *in silico*-identified Inc proteins in *C. psittaci* 02DC15, including orthologous loci of *C. psittaci* VS225 and *C. psittaci* 6BC. Total size and sizes of relevant Inc domains are given for each protein; aa, amino acids; TMα; transmembrane α-helix.

	*C. psittaci* 02DC15							*C. psittaci* VS225	*C. psittaci* 6BC
	Accession number CP002806.1							Accession number CP003793.1	Accession number CP002549.1
Name	Locus	Total/aa	N-terminal domain/aa	1st TMα/aa	loop/aa	2nd TMα/aa	C-terminal domain/aa	Locus	Locus
Cps0558	CPS0B_0558	372	36	22	5	24	285	B600_0596	CPSIT_0555
Cps0181	CPS0B_0181	1012	48	22	5	19	918	B600_0189	CPSIT_0179
Cps0355	CPS0B_0355	224	36	25	5	24	134	B600_0375	CPSIT_0350
Cps0855	CPS0B_0855	810	44	26	6	23	711	B600_0910	CPSIT_0848
Cps0856	CPS0B_0856	342	28	27	6	21	260	B600_0911	CPSIT_0850
Cps0849	CPS0B_0849	238	26	25	5	22	160	B600_0904	CPSIT_0841
Cps0857	CPS0B_0857	479	32	21	6	22	398	B600_0912	CPSIT_0851
IncA^a^	CPS0B_0598	382	67	24	5	21	265	B600_0636	CPSIT_0594
IncB^b^	CPS0B_0535	202	118	32	11	25	16	B600_0570	CPSIT_0532
IncC^b^	CPS0B_0534	184	103	23	19	24	15	B600_0569	CPSIT_0531
Cps0850^c^	CPS0B_0850	352	26	26	11	24	265	B600_0905	CPSIT_0842

a Rockey et al. (1995).

b Bannantine et al. (1998).

c Huang et al. (2023) and Xiao et al. (2023).

### Immunofluorescence imaging confirms Cps0558 as bona fide Inc protein

**Figure 2.**
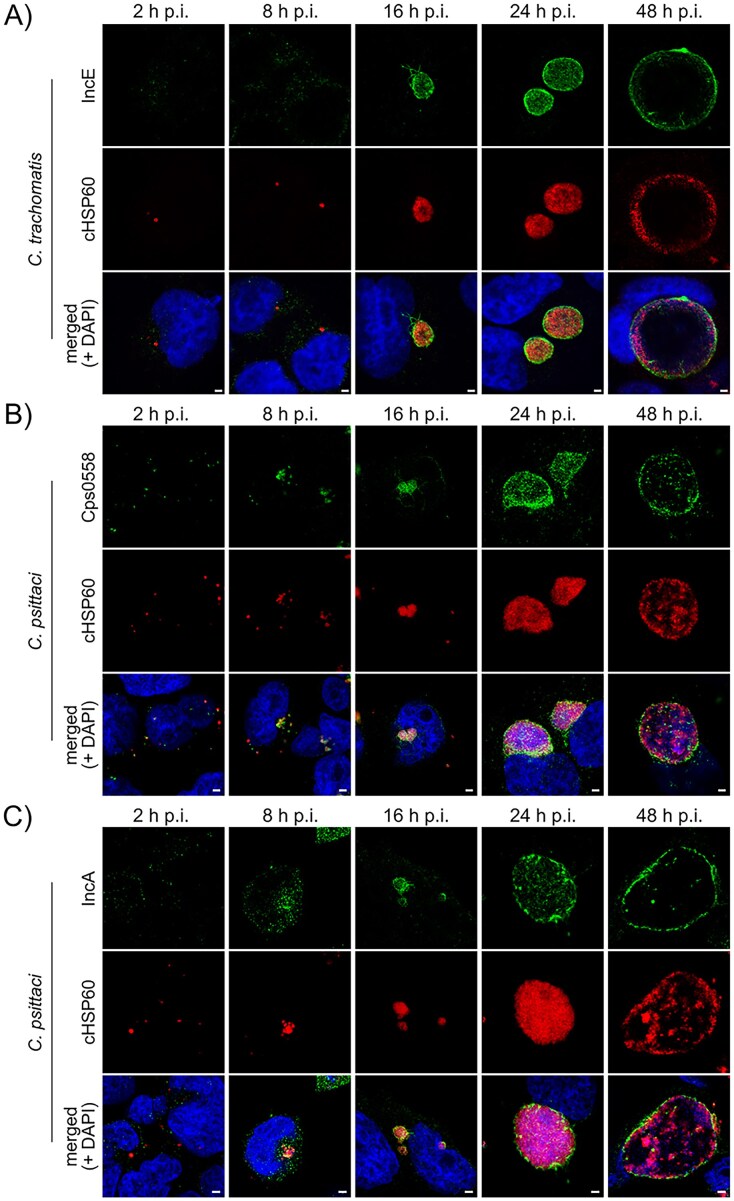
Time course analysis of the expression and subcellular localization of Cps0558 and IncA during the *C. psittaci* infection cycle. Representative immunofluorescence images of *C. psittaci*- and *C. trachomatis*-infected HeLa cells (MOI 2) at 2, 8, 16, 24, and 48 h p.i., captured using a laser scanning confocal microscope. Cells were fixed with PFA and *C. trachomatis* IncE (A), *C. psittaci* Cps0558 (B), or *C. psittaci* IncA (C) were detected using a rabbit-anti-IncE antibody, rabbit-anti-Cps0558 antibody, or a rabbit-anti-IncA antibody, respectively. cHSP60 was detected using a mouse-anti-cHSP60 antibody and DNA was counterstained using DAPI. All micrographs are displayed at the same magnification for the respective time points. Scale bar, 2 µm; *n* = 3.

Inc proteins typically display a characteristic rim-like staining pattern, which is indicative of their localization within the inclusion membrane. To confirm the localization and validate the predicted *C. psittaci* Inc proteins in comparison to *C. trachomatis* IncE, we infected HeLa cells with *C. psittaci* or *C. trachomatis* and analysed their timing of expression and subcellular localization at specific time points during infection (2–48 h p.i.) using immunofluorescence imaging (Fig. 2).

As previously shown, IncE of *C. trachomatis* was first detected at 16 h p.i. and localized in the characteristic rim-like staining pattern around the inclusion (Fig. 2A). This pattern remained unchanged until 48 h p.i. Interestingly, IncE-positive tubule-like structures were observed extending outward from the from the inclusion membrane into the host cytoplasm at 16 h p.i. For further analysis of *C. psittaci* Inc proteins, we focused on Cps0558 and IncA. Clear detection of both Cps0558 and IncA started at 8 h p.i. (Fig. 2B and C). While IncA localized in the inclusion membrane starting at 16 h p.i. (Fig. 2C), Cps0558 localized within the inclusion until 16 h p.i. and shows a characteristic rim-like staining pattern only at 48 h p.i. (Fig. 2B). Starting at 16 h p.i., Cps0558 and IncA were also present in tubular-like structures emanating from the inclusion (Fig. 2B and C), a phenotype already described for *C. psittaci* IncA (Rockey et al. 1995). Although Cps0558 expression was detected early during infection, the characteristic rim-like phenotype was only observed at 48 h p.i. To determine Cps0558 sublocalization between 24 and 48 h p.i. in detail, we acquired z-stacks of HeLa cells infected with *C. psittaci* at 32 h p.i. (Fig. S2). Surprisingly, Cps0558 was fully localized in the inclusion membrane and tubular-like structures, with no detectable signal in the inclusion lumen.

Taken together, these findings confirm that *C. psittaci* IncA and the predicted Inc protein Cps0558 are indeed bona fide Inc proteins, localizing in the inclusion membrane, similar to the previously characterized *C. trachomatis* IncE.

### Inc/GFP chimera protein-based interactomics is suitable for detection of relevant *in vivo* Inc protein interactions

**Figure 3.**
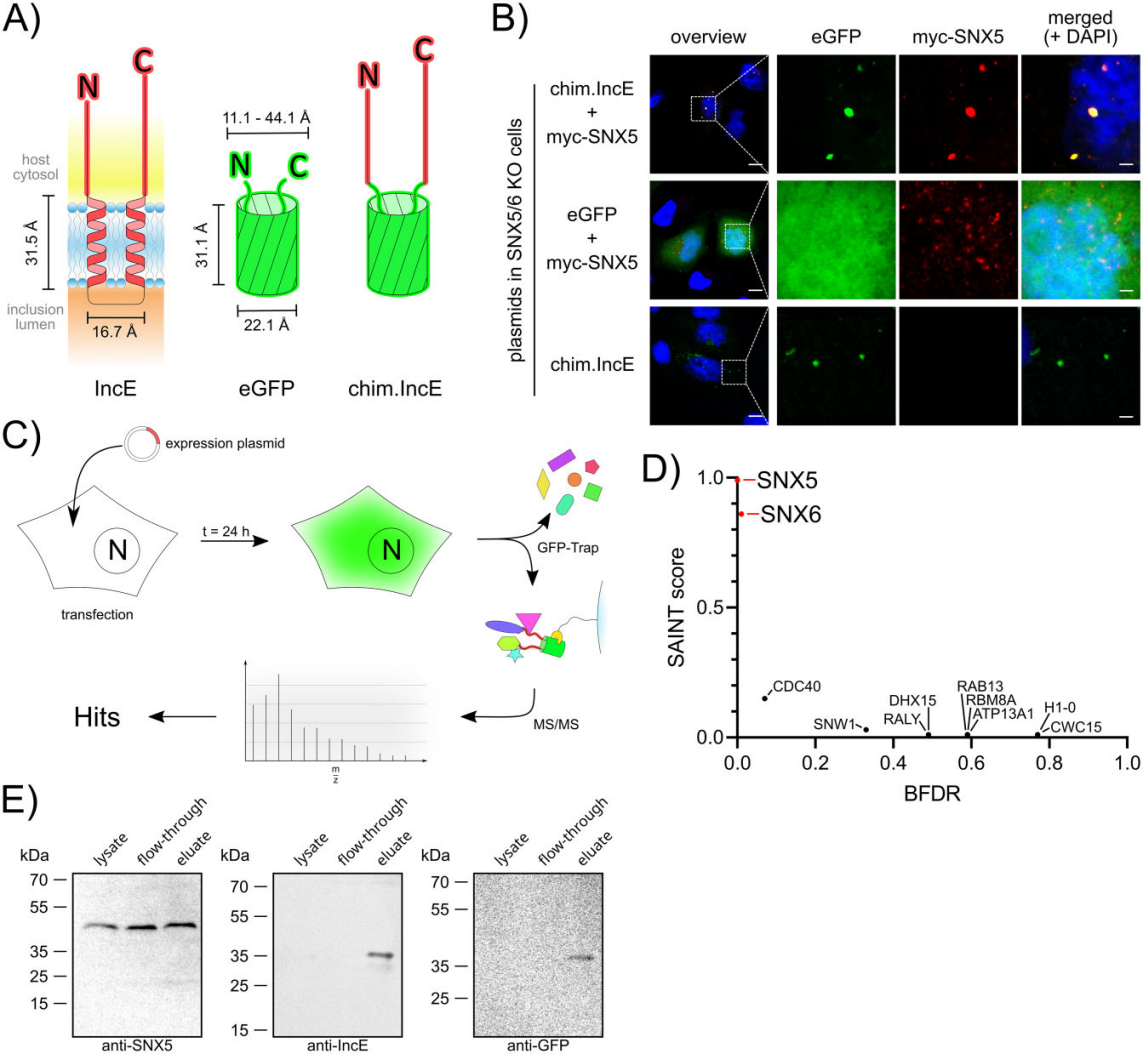
Novel Inc/GFP chimera protein-based approach and its functional validation. (A) Schematic representation and design of Inc/GFP chimera proteins. The scheme illustrates topology and proportions of IncE (*C. trachomatis*), eGFP, and the fusion protein chim.IncE. Atomic distances (CA) were estimated using PyMol (IncE, Uniprot ID: P0DJI4; eGFP PDB: 2Y0G). The lengths of the cytosolic domains of IncE are represented relative to each other. (B) Immunofluorescence images showing colocalization of chim.IncE and myc-SNX5. HeLa cells with SNX5 and SNX6 double-knockout were transfected with different plasmids for the expression of chim.IncE and myc-SNX5, chim.IncE alone, or eGFP alone (left). After 24 h, cells were fixed with PFA and stained using a mouse-anti-myc antibody and DAPI (DNA). Images were captured using a laser scanning confocal microscope. Scale bar: overview, 10 µm; zoom, 2 µm; *n* = 3. (C) Workflow of the Inc/GFP chimera protein interactomics approach. HeLa cells are transfected with a plasmid encoding for specified Inc/GFP chimera proteins. After 24 h, cells are lysed, and the Inc/GFP chimera protein, along with interacting proteins, is enriched using the GFP-Trap kit (Chromotek). Enriched proteins are directly digested on beads with trypsin, followed by desalting through Stage-Tips and LC–MS analysis. Statistically enriched hits are identified using SAINT (Olson et al. 2020). (D) Interaction partners of chim.IncE. Interaction partners were identified following the workflow outlined in (C) and are plotted as SAINT score versus BFDR. SAINT scores were calculated using the control eGFP (*n* = 1). Known IncE interaction partners are indicated in the plot. The complete list of identified proteins is provided in Table S1. (E) Western blot analysis of GFP-Trap pulldown targeting chim.IncE. HeLa cells were transfected with a chim.IncE expression plasmid, lysed after 24 h, and subjected to GFP-Trap pulldown. Input (lysate), flow-through, and elution fractions were analysed by SDS-PAGE and western blotting using anti-GFP, anti-IncE, and anti-SNX5 antibodies.

Next, we aimed to identify potential protein interactions of IncA and Cps0558 with host cell proteins. In the past, the identification of Inc protein interaction partners was challenging due to the low solubility of the TM domain (Subtil et al. 2001). This low solubility likely hampers standard techniques for identifying interaction partners, such as pull-down assays. Additionally, studies using only one soluble terminus could miss some interaction partners (Mirrashidi et al. 2015, Kumagai et al. 2018). To overcome these problems, we replaced the TM domains with enhanced green fluorescent protein (eGFP) thus forming a soluble protein that contains both split termini (Inc/GFP chimera protein, or chim.Inc protein for short) and assumably enhances solubility and binding of potential interaction partners (Fig. 3A). Notably, eGFP has similar dimensions as the replaced TM domains and the luminal loop of the Inc proteins. The fusion protein further simulates the topology of Inc proteins inserted into the inclusion membrane by placing both termini at the same side of the eGFP barrel structure.

For proof-of-concept, we again used the well-studied Inc protein IncE from *C. trachomatis*, which is known to interact with host cellular sorting nexins SNX5 and SNX6 (Mirrashidi et al. 2015, Elwell et al. 2017, Paul et al. 2017, Sun et al. 2017). The eGFP fusion protein of IncE, termed chim.IncE, was expressed in HeLa cells with a double knockout of SNX5 and SNX6 together with a myc-tagged version of SNX5 (Fig. 3B). Upon immunofluorescence staining of myc-SNX5, chim.IncE showed a punctate subcellular distribution and indeed fully colocalized with myc-SNX5. In contrast, control conditions using either GFP alone or no coexpression of myc-SNX5 consequently showed no colocalization of both fluorescence signals.

Next, we used chim.IncE to establish and validate an experimental workflow for identification of Inc protein interaction partners (Fig. 3C). Here, HeLa cells were transfected with the chim.IncE expression plasmid and after 24 h, IncE and interacting proteins were enriched via GFP-Trap pulldown of chim.IncE followed by mass spectrometry. Prior to mass spectrometry analysis, we confirmed expression and integrity of the chimera by western blotting (Fig. 3E), detecting a single band of the expected size with both anti-IncE and anti-GFP in the elution fraction, indicating no degradation. Importantly, endogenous SNX5 was detected in the elution fraction, confirming specific interaction with chim.IncE. Following this validation, we performed mass spectrometry to identify interaction partners. Statistically significant hits were determined using the spectral count-based SAINT algorithm (Olson et al. 2020). Strikingly, we were able to identify SNX5 and SNX6 as the most highly enriched interaction partners in the pulldown fraction (Fig. 3D). Apart from that, another nine weakly enriched proteins were detected. Taken together, these results demonstrate that Inc/GFP chimera proteins are a validated and valuable tool to detect relevant Inc protein interactions.

### Inc/GFP chimera protein-based interactomics reveals novel host cellular interactions of Cps0558 and IncA

**Figure 4.**
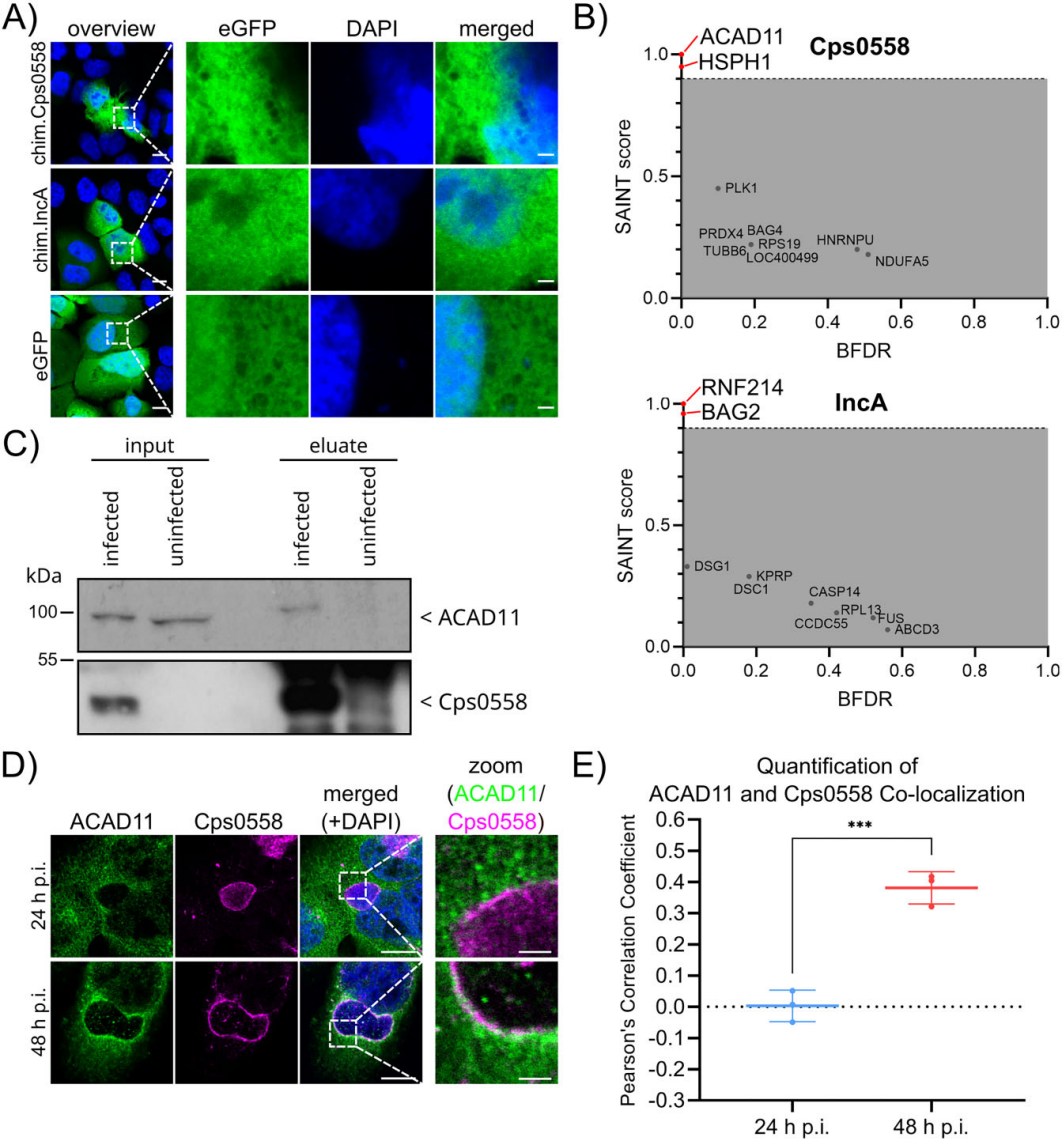
Microscopic and proteomic analysis of Inc/GFP chimera proteins Cps0558 and IncA. (A) Subcellular localization of Inc/GFP chimera proteins and eGFP. HeLa cells were transfected with the plasmid encoding the protein of interest, as indicated on the left, for 24 h and subsequently fixed with PFA. DNA was stained with DAPI. The eGFP segment of the expressed construct is shown. Images were taken using a laser scanning confocal microscope. Scale bar: overview, 10 µm; zoom, 2 µm; *n* = 3. (B) Interaction partners of chim.Cps0558 and chim.IncA. Interaction partners were identified following the workflow outlined in Fig. 3(C) and are plotted as SAINT score versus BFDR. The SAINT score of each sample was calculated using all other samples as controls (biological triplicates). Significant proteins were determined with SAINT score ≥0.9 and BFDR <0.01. The top 10 proteins with the highest SAINT scores for each bait are shown, while the complete list of identified proteins is provided in Tables S2–S4. (C) Interaction partner of endogenous Cps0558. HeLa cells were left uninfected or infected with *C. psittaci* and lysed at 46 h p.i. Lysates were subjected to immunoprecipitation using an antibody against Cps0558. Input (postnuclear supernatant), and elution fractions were analysed by SDS-PAGE and western blotting using anti-Cps0558 and anti-ACAD11 antibodies. (D) Immunofluorescence images of ACAD11 and Cps0558 in *C. psittaci*-infected HeLa cells. Cells were fixed at 24 and 48 h p.i. with PFA and stained using mouse-anti-ACAD11, rabbit-anti-Cps0558 and DAPI (DNA). Scale bar: overview, 10 µm; zoom, 2 µm; *n* = 3. (E) Quantification of colocalization between ACAD11 and Cps0558 based on the immunofluorescence images shown in (D). Pearson’s correlation coefficients were calculated using ImageJ (Fiji) with the Coloc2 plugin. Data represent the mean ± SD from *n* = 3 biological replicates, based on 35–58 inclusions (24 h p.i.) and 26–43 inclusions (48 h p.i.) per biological replicate. Statistical significance was determined using an unpaired two-tailed Student’s *t*-test. ***P < .001.

Building on the successful validation of our Inc/GFP chimera protein-based approach, we next applied this method to further characterize the validated Inc proteins Cps0558 and IncA. Thus, we generated the corresponding fusion proteins chim.Cps0558 and chim.IncA. To analyse their subcellular localization and confirm their expression, we first transfected uninfected HeLa cells with plasmids encoding either the Inc/GFP chimera protein or eGFP as a control, and counterstained with DAPI 24 h posttransfection (Fig. 4A). As with the eGFP control, all Inc/GFP chimera proteins showed a distribution throughout the cell including the nucleus, showing that they are soluble. Next, we continued with the established pulldown approach of the Inc/GFP chimera proteins to identify potential interactions. Uninfected HeLa cells were transfected with a plasmid encoding the respective Inc/GFP chimera protein and lysed 24 h posttransfection for pulldown via GFP-Trap and subsequent mass spectrometry (Fig. 4B, Tables S2–S4). Proteins with a SAINT score ≥0.9 were defined as significantly enriched. For Cps0558 (top), we identified Acyl-CoA dehydrogenase family member 11 (ACAD11) and Heat shock protein 105 kDa (HSPH1) as significantly enriched, while all other identified proteins had SAINT scores below 0.5. For IncA, the RING finger protein 214 (RNF214) and BAG family molecular chaperone regulator 2 (BAG2) were significantly enriched in the interactome (bottom). For further validation of our Inc/GFP chimera protein-based approach, we focused on the interaction between Cps0558 and ACAD11. To directly confirm this interaction in the context of infection, we performed immunoprecipitation using an antibody against Cps0558 from *C. psittaci*-infected or uninfected HeLa cells, which served as negative control, followed by western blot analysis. ACAD11 was specifically detected in both the input and eluate of infected cells, whereas in uninfected cells, ACAD11 was only present in the input (Fig. 4C). These findings both demonstrate that ACAD11 coprecipitates with Cps0558 from infected cells and provide strong biochemical evidence for a physical interaction under native conditions. We next analysed the subcellular localization of these two proteins during the infection (Fig. 4D). We performed immunofluorescence analysis of ACAD11 and Cps0558 at 24 and 48 h p.i. in *C. psittaci*-infected HeLa cells. At 24 h p.i. ACAD11 staining demonstrated a diffuse staining pattern typically for cytosolic proteins. Interestingly, at 48 h p.i. ACAD11 staining strongly accumulated at the inclusion membrane, temporally coinciding with detection of Cps0558 in the characteristic rim-like staining pattern indicative of inclusion membrane localization (Fig. 4D). Notably, partial overlap of ACAD11 (green) and Cps0558 (magenta) signals was observed at the inclusion membrane, visible as white signals. We further recorded z-stacks of HeLa cells infected with *C. psittaci* at 24 and 48 h p.i and analysed the colocalization of ACAD11 and Cps0558 (Fig. S3A and B). The resulting orthogonal confocal projections confirm our 2D observations. At 48 h p.i., ACAD11 accumulates at the inclusion membrane and overlaps with the Cps0558 signal, independent of the curvature of the plasma membrane. In contrast, at 24 h p.i., ACAD11 remains diffusely cytosolic and shows no specific localization, while Cps0558 signal was also detectable in the inclusion lumen. To quantify the colocalization of ACAD11 and Cps0558 from Fig. 4(D), we performed a quantitative image analysis using the Pearson’s correlation coefficient (Fig. 4E). The Pearson’s coefficient, ranging from −1 (mutual exclusion) to +1 (perfect colocalization), increased significantly from 0.003 ± 0.05 at 24 h p.i. to 0.3812 ± 0.0519 at 48 h p.i., indicating the interaction of ACAD11 and CPS0558 at the inclusion membrane during late stages of infection. Taken together, these results demonstrate that our Inc/GFP chimera protein-based approach is capable of identifying novel host cellular interactions of *C. psittaci* Inc proteins, thus paving the way for in-depth analyses of their function and relevance in the context of *C. psittaci* infection.

## Discussion

The chlamydial inclusion membrane, which serves as interface between pathogen and host, is essential for successful chlamydial development. Numerous Inc proteins identified for *C. trachomatis* have been shown to drive host cell manipulation, subsequently benefiting chlamydial growth (Bugalhão and Mota 2019). While research on host–pathogen interactions of *C. trachomatis* has progressed over decades, little is known about *C. psittaci* and its Inc proteins. The lack of classical sequence motifs of Inc proteins limits the identification of novel Inc proteins, and their integral localization in the inclusion membrane further complicates experimental approaches, as membrane proteins are challenging to isolate and study without disrupting their native environment. To address these limitations, we performed a genome-wide *in silico* screening for identification of Inc proteins and established a novel method, the Inc/GFP chimera protein-based interactomics, to facilitate *in vivo*-characterization of the identified proteins and their interactions.

*In silico* analyses based on hydrophobicity prediction have been reported to accurately determine TM domains of potential Inc proteins and are still used today (Rockey et al. 1995, Bannantine et al. 1998, Lutter et al. 2012, Xiao et al. 2023). Applying this approach, we screened the genome of *C. psittaci* 02DC15 for both previously annotated Inc proteins and hypothetical proteins with unknown function, identifying 60 candidate Inc proteins based on the presence of two predicted TM domains. Cross-referencing with existing datasets revealed that 47 of the 60 proteins had predicted orthologs in *C. caviae* (Dehoux et al. 2011) and 6 in *C. trachomatis* (Pereira et al. 2024), supporting their potential functional conservation within the genus. To focus our downstream analyses, we prioritized proteins that were computationally preannotated as Inc proteins, yielding a subset of 11 for further analysis. We validated TM domains in all 11 Inc proteins identified in our study, which included four previously characterized and seven newly proposed candidates. Another recent genomic screen also reported the identification of 11 Inc proteins in *C. psittaci*, of which nine overlap with our subset (Sachse et al. 2023). Typically, putative Inc proteins are confirmed *in vivo* with specific antibodies in immunofluorescence assays of cells infected with *Chlamydia* spp., where they show a rim-like pattern (Bannantine et al. 1998, Weber et al. 2015). In our immunofluorescence analysis, we validated the predicted Inc protein Cps0558 as a novel bona fide Inc protein based on its localization in the inclusion membrane similar to IncA and IncE from *C. trachomatis*. IncA and Cps0558 showed a rim-like localization with tubular structures, consistent with previous reports for *C. psittaci* IncA, *C. trachomatis* IncA, IncG, and IncE (Rockey et al. 1995, Bannantine et al. 1998, Rzomp et al. 2003, Mirrashidi et al. 2015). The formation of such tubular structures in *C. trachomatis* has been discussed to be connected with IncE and its recruitment of host cellular retromer components (Aeberhard et al. 2015, Mirrashidi et al. 2015). As *C. psittaci* lacks an IncE orthologue, an additional mechanism remains to be investigated. These structures may facilitate interactions with host cell organelles or contribute to the organization of the inclusion environment, potentially supporting chlamydial growth and development.

To assess possible functions of Cps0558 and IncA, we characterized and categorized expression and localization of the investigated Inc proteins during *C. psittaci* infection from 2 to 48 h p.i. Based on the detection at 8 h p.i., we classified Cps0558 as a mid-phase Inc protein and confirmed IncA as a mid-phase Inc protein, consistent with its previous classification (Rockey et al. 1995). Early-phase Inc proteins, such as *C. trachomatis* IncD, IncE, IncF, and IncG, support the development and establishment of the chlamydial inclusion (Scidmore-Carlson et al. 1999), while mid-phase Inc proteins such as CT813 participate in maintaining the inclusion integrity (Wesolowski et al. 2017). This suggests similar functions for Cps0558 and IncA. In general, Inc proteins are expressed in the bacterial cytosol and translocated into the inclusion membrane via T3SS (Subtil et al. 2001). The observations made in the present study revealed an initial intracellular signal for IncA, detected in the bacterial cytosol at 8 h p.i. and at 16 h p.i. a rim-like staining pattern was detected, characteristic of Inc proteins. This temporal delay in membrane localization could be indicative of either a lag in secretion dynamics or the need for protein accumulation to reach detectable levels at the inclusion membrane. The subsequent decrease in the intracellular signal suggests that the expression of IncA is temporally regulated and ceases as the infection progresses. Cps0558 was detected at 8 h p.i. in the bacterial cytosol and a typical rim-like staining pattern surrounding the bacterial inclusion was first seen 24 h p.i. by indirect confocal immunofluorescence microscopy, becoming more distinct at 32 h p.i. This temporally delayed membrane localization, despite early expression, suggests that Cps0558 may become functionally relevant at the membrane later than other mid-phase Inc proteins such as IncA.

Investigations of Inc proteins and their interactions are challenging. Initial approaches of ectopically expressed full-length Inc proteins resulted in their mislocalization due to their TM domains (Basovskiy et al. 2008, Li et al. 2008, Shkarupeta et al. 2008). Subsequent studies focused solely on the cytosolic C-terminal domains (Böcker et al. 2014, Mirrashidi et al. 2015, Saka et al. 2015, Huang et al. 2023), which renders Inc proteins soluble while potentially influencing both their folding and their interactions. This limitation is evident in *C. trachomatis* IncD. Interaction partners were not identified when only the C-terminal domain of IncD was expressed (Mirrashidi et al. 2015). In contrast, it was demonstrated that the interaction with the host cellular protein CERT requires both the N- and C-terminal domains of IncD (Kumagai et al. 2018). These findings highlight that the exclusion of essential structural domains, such as the N-terminus, can significantly affect the ability to detect interaction partners, leading to incomplete or misleading conclusions about the protein’s functional interactions. Furthermore, proximity interactomics using APEX2-tagged Inc proteins in genetically modified *Chlamydia* enables identification of surrounding host factors (Rucks et al. 2017, Dickinson et al. 2019), but does not resolve direct from indirect interactions. In addition, C-terminal tagging may interfere with native binding, and the need for genetic manipulation poses biosafety and regulatory challenges, especially for zoonotic strains like *C. psittaci*.

To address these challenges, we introduced a novel Inc/GFP chimera protein-based interactomics approach. The replacement of the native TM domains with eGFP in Inc/GFP chimera proteins enables the cis direction of both cytosolic termini in close proximity, thereby approximating the native state of Inc proteins. Furthermore, the solubilization of the protein allows for unrestricted interaction with host cellular proteins. Ultimately, the eGFP portion facilitates immunofluorescent analysis and coimmunoprecipitation for proteomic studies of interaction partners. We used the well-studied IncE from *C. trachomatis* as positive control to establish the novel approach. In immunofluorescence microscopy, the eGFP-containing chim.IncE appeared punctate, both in the presence and absence of its known interaction partners. While this phenotype can be aligned to interactions with SNX5, which simultaneously binds chim.IncE and the punctate retromer complex, the punctate distribution observed in the absence of these known interactions requires further explanations. Punctation via homooligomerization can be excluded, as the blank eGFP control does not display this phenotype and native IncE is known not to form homooligomers (Gauliard et al. 2015). Artifacts of degraded expression products can also be excluded, as western blot analysis showed no evidence of fragmentation of chim.IncE with either anti-IncE or anti-GFP antibodies. Alternatively, this phenotype could result from weak interactions between chim.IncE and other retromer components, independent of SNX5 and SNX6. This hypothesis is supported by our proteomics data. While SNX5 and SNX6 were validated as interaction partners, we also identified nonsignificantly enriched proteins. We suggest that they derive from secondary interactions, but we cannot exclude them as potential additional IncE interactions. Future interaction studies in SNX5 and SNX6 double-knockout cells could reveal potential alternative interaction partners of IncE.

Interestingly, for IncA, we detected two novel interaction partners, RNF214 and BAG2, which are involved in protein quality control. RNF214 exerts the function of an E3 ubiquitin ligase (Barroso-Gomila et al. 2023) critical for nonproteolytic ubiquitylation (Lin et al. 2024), while BAG2 is involved in ubiquitin-independent stress routes to proteasomal degradation (Carrettiero et al. 2009, 2022). It is well-known that *Chlamydia* spp. interact with and manipulate host ubiquitylation machinery. For instance, pulldown assays have demonstrated the copurification of ubiquitylation proteins with *C. trachomatis* Inc proteins (Mirrashidi et al. 2015). Recently, the Inc protein CT_135 (GarD) from *C. trachomatis* has been shown to prevent ubiquitylation by the E3 ubiquitin ligase domain-containing RNF213 (Walsh et al. 2022). This protein is induced by IFN-γ and is known to initiate ubiquitination of viruses, *Toxoplasma gondii* and *Salmonella* in infected cells (Otten et al. 2021, Thery et al. 2021, Hernandez et al. 2022, Tian et al. 2023, Zhang et al. 2023). Since there is no orthologue of CT_135 in *C. psittaci*, IncA might function similarly. Additionally, chlamydial deubiquitylases were shown to inhibit ubiquitylation of Inc proteins, the inclusion, NF-κB, and Mcl1, thereby preventing inclusion degradation, inflammatory response, and host-induced apoptosis (Haldar et al. 2016, Wang et al. 2017, Ramirez et al. 2018, Bastidas et al. 2024). As chlamydial development progresses and host stress increases, the host degradation machineries become crucial in the late phase and might explain the mid-phase expression of IncA, suggesting its role in host ubiquitylation manipulation. Notably, our results differ from the reported interaction partner G3PB1 (Borth et al. 2011). While strong interactions such as *C. trachomatis* IncE with SNX5/SNX6 showed distinct colocalization at the inclusion membrane (Mirrashidi et al. 2015), the recruitment of G3PB1 was demonstrated as accumulation in the proximity of the inclusion (Borth et al. 2011). Since we did not find any peptide fragment aligning with G3BP1 or similar proteins, we suggest that the reported interaction between G3BP1 and IncA is not strong enough for detection in our approach. Further investigations and direct interaction studies are necessary to determine mechanisms and functions of IncA.

Our novel Inc/GFP chimera protein-based interactomics approach revealed ACAD11 as an interaction partner of the Inc protein Cps0558. ACAD11, a key enzyme in the alternative β-oxidation of very-long-chain fatty acids (≥20 carbon atoms) in peroxisomes (He et al. 2011, Camões et al. 2015), plays a critical role in lipid metabolism. *Chlamydia* spp. are known to scavenge host cellular lipids (Wylie et al. 1997, van Ooij et al. 2000, Heuer et al. 2009) and recruit related host proteins to the inclusion, such as the ceramide transport protein CERT in *C. trachomatis* and *C. psittaci* (Derré et al. 2011, Koch-Edelmann et al. 2017), and long-chain acyl-CoA synthetases in *C. trachomatis* (Recuero-Checa et al. 2016). Therefore, we hypothesize that the recruitment of ACAD11 via Cps0558 similarly targets the host cellular lipid metabolism and facilitates the local uptake of such lipids. Given its uniquely prolonged localization pattern, Cps0558 might play a role in the lipid acquisition strategy of *C. psittaci* during the late phases of its development. This interpretation is further supported by our time-resolved immunofluorescence analysis, which revealed that ACAD11 is recruited to the inclusion membrane only at late infection stages. Quantitative analysis of Cps0558 and ACAD11 colocalization by Pearson’s correlation coefficient showing a moderate but significant increase in Pearson’s correlation coefficient from 24 to 48 h p.i., independent of plasma membrane curvature at late stages of infection. In addition, we confirmed this interaction under native infection conditions through coimmunoprecipitation of Cps0558 followed by western blot, detecting ACAD11 as a specific interactor during infection. Together, these complementary biochemical and imaging data support a biologically relevant interaction between ACAD11 and Cps0558. In contrast to IncE and SNX5/6 for example, which exhibit near-complete colocalization (Paul et al. 2017), the partial recruitment of ACAD11 may be explained by its endogenous distribution to the cytosol and vesicular compartments (He et al. 2011), limited availability of specific isoforms, or restricted and transient interactions at the inclusion. We hypothesize that the membrane-proximal recruitment of ACAD11 by Cps0558 during late infection supports inclusion maintenance while preserving essential host cell functions, thereby promoting prolonged host cell survival.

Our study highlights the suitability of the newly established Inc/GFP chimera protein-based interactomics approach as a valuable tool to perform interaction studies between Inc proteins and host cell proteins. Our results contribute to filling the gap of Inc protein research for *C. psittaci* and support future interactome studies.

## Supplementary Material

ftaf012_Supplemental_Files
